# Treatment of Tetanus

**Published:** 1862-08

**Authors:** J. F. Norton

**Affiliations:** Fort Edward


### ART. IV.—*Treatment of Tetanus.* *By* J. F. Norton, M. D.

Fort Edward, June 20th, 1862.

While looking over my notes upon practice, taken in south-western Georgia, within the last three years, and up to the commencement of the present rebellion, my attention is particularly called to the successful treatment of some five cases of tetanus, by equal proportions of chloroform and sulphuric ether.

Thinking perhaps a brief detail of the treatment of these cases might be of some service to many readers of the "Buffalo Medical and Surgical Journal," I take great pleasure in presenting them through its medium. There can exist no doubt but this formidable affection is a disease peculiar to a tropical climate, where it is no respecter of persons, age, nor sex, except, perhaps, it may be more prevalent among the negro than the white population; and this, I think, arises not so much from constitutional predisposition as from their greater exposure to wounds of the feet, and bites of venomous insects, &c.

The profession have long been laboring to find a reliable remedy or cure for this disease, but, alas! have failed, except, perchance it may prove to be the ones now under consideration. Indeed I am aware that chloroform has already found great favor with many able authors, as a cure for tetanus, and previous to my visit South I well knew that it had its admirers and supporters, while many others, together with some of our schools and hospitals, as earnestly denounced it as unsafe, and particularly unwarrantable in its use, in all cases of a tubercular diathesis. Whatever distrust I might have entertained of its almost indiscriminate use in some of our Northern hospitals, I soon lost it in the South, where I found it not only used by all physicians, but by the planters themselves, whose medicine chests as invariably contained chloroform as they did quinine.

Case 1st—Was that of a young negro who came into town during the day with a load of cotton; while at the ware-house he was observed to fall from a cotton bale upon which he was sitting, and was supposed to be in a dying condition. I saw him in twenty minutes later, found him *opisthotnos;* his body curved, and resting upon the heels and back of head, eyes partially closed, pupils contracted, breast heaving laboriously, with a rhoncus rattle of the chest and throat. His arms were drawn up and rigid with spasm, a mere fluttering of the pulse, the joints firmly closed, trismus complete. Being unable to get anything down him, I hastened to procure a mixture of chloroform and sulph. ether, equal parts. I returned in ten minutes; he had been removed to a room; the mucous rattle now seemed to threaten suffocation; while quantities mixed with blood was forced from his mouth and nose. I instantly applied the sponge to his nose, saturated with perhaps two drachms of the ether mixture. The first effect was a sudden convulsive movement of the whole body, which soon lost its curvature, and dropped upon the floor; the rattling and heaving of the breast began to abate, and the arms fell powerless by his side. In less than five minutes he seemed entirely relieved, and his eyes appeared closed in heavy sleep. I now found the trismus sufficiently yielding to enable me to get down twenty grains calomel and five morphine. I withdrew the sponge, occasionally re-applying it for few seconds at a time, until all symptoms of spasm had disappeared, he sleeping soundly. The breathing was now free from rattle, the inspirations slow, and full; pulse distinct and firm. I left him with orders to get oil and turpentine in equal parts in four hours. I saw him the following morning. The oil had passed off freely. He had no recollection of what had passed, felt weak, otherwise quite well. He returned home, and speedily recovered, upon tonics and anodynes.

2d case—Was a white man, aged forty years, who eight days previous received the contents of a musket, loaded with buck-shot in his right side, arm and hand. Two of his fingers were amputated by his attending physician. I found the man looking ghastly pale, eyes sunken, the pulse 140, low and tremulous. The whole body was bathed in a clammy sweat. He tried to articulate, but could not, nor could he swallow, the masticatory muscles were rigid. He had complained, the day previous, of stiffness of the neck, burning sensation in the throat, with some difficulty in swallowing, pain and twitching of the wounded arm and hand. These symptoms continued to increase until they presented all the phenomena of traumatic-tetanus. His bowels were tender under the hand. The wounds presented an ashy, dry, gangrenous look. I soon brought him under the influence of the ether mixture, and with the happiest results. The breathing which was before hurried and difficult, now became tranquil, pulse firmer and less frequent; in fact the tetanic symptoms gradually subsided. The masticatory muscles having become sufficiently relaxed, I soon administered my *favorite* prescription, twenty grains calomel, with all the morphine I thought he could bear. I soon left him sleeping, with instructions to give oil and turpentine, equal parts, early the following morning, it being now night, and should the spasms return, to call me. When I saw him in the morning the bowels had been relieved of copious, dark and offensive evacuations, and much of the tenderness. I now gave him quinine and wine, together with anodynes. The day following he was stronger, could swallow well, The wounds looked brighter, and were moist, with healthy suppuration.

On the fourth day, being the eleventh from the accident, he complained of slight stiffness of the neck, with burning in the throat. Being fearful he might get a return of spasms, I ordered him, through the day, teaspoon-full doses of the ether mixture; this dispelled all tetanic symptoms, and they did not return. He recovered upon tonics and anodynes.

My third and fourth cases were blacks, which I will not detail at length, merely stating that they both proceeded from incised wounds; one of the head, the other of the foot. They both recovered with no alterations in the previous plan of treatment, except the administration of croton oil, in the latter case, owing to great torpidity of the bowels.

Fifth, and last case, was a white man, aged forty years, of intemperate habits. He received a wound in the hand while attempting to grasp a drawn knife from his adversary. The superficial palmar arch was divided, from which he had lost much blood before I saw him. I succeeded in securing the vessel, by compression upon both ends. The following day I was called in haste, the messenger stating, to use his language, that his master "had gone ded in de fit." Upon coming to his bedside, he appeared more like one in a heavy nightmare, contracted features, eyebrows and fore-head corrugated, jaws firmly closed, the general expressions were changa-ble, sometimes presenting the true *risus sardonicus* of authors. his limbs were rigid, and the eyes sunken, pupils contracted, pulse very feeble. He was soon brought under the influence of the mixture, when the face quickly lost its convulsive features, and the tetanic symptoms began to mitigate, and in three minutes he appeared sleeping. The trismus in this case. however, did not yield as readily as those previous, but the cervical and masticatory muscles gradually gave way, and I gave him twenty grains calomel and morphine, all he could bear; this, as usual, was fol-lowed with oil and turpentine at proper time, which passed off freely relieving him. He complained on the following day of slight twitching of the wounded hand, with cramping; this was relieved by a few doses of the mixture, taken internally. I ordered him quinine with wine. In a few days he was fully restored.

Since my return North I have been in the habit of using chloroform and ether in convulsions of children, and with the best of success. I have seen also, tetanus cured in two horses. I merely state these cases to show my experience in the use of these articles.

In conclusion, I will remark first, that the great difficulty in the way of curing tetanus, is trismus. 2d; That chloroform and sulph. ether will, if properly administered, sufficiently relieve the spasmodic contraction of jaws and throat, to enable the physician to employ internal remedies. 3d; I have no doubt that these valuable anæsthetics will, of themselves, cure most, if not all, cases of this disease, by their judicious use, both by inhalation and internal administration. 4th; That the most reliable or effectual plan of treatment, is to assist them by internal remedies as soon as they can be given, and these remedies are calomel and morphine, in full doses, followed with oil and turpentine; afterwards the tonics and anodynes, quinine and morphine.

The chloroform and ether should be used in equal proportions, to be successful, and administered without fear; first, knowing the patients constitution; and secondly, what we want to effect with it. *

* NOTE.—The conclusions of the author do not correspond fully with the experience of physicians at the North who have used anæsthetics in tetanus. We cannot regard the value of calomel as established; and have recently seen a child suffer the severest tetanic spasms while yet fully under the influence of chloroform. We most fully agree in the use of morphine and quinine, and have no doubt as to the value of anæsthetics; the experience of the author is very satisfactory, in the use of chloroform and ether in combination.—[Ed.

